# Rethinking Schizophrenia in the Context of the Person and Their Circumstances: Seven Reasons

**DOI:** 10.3389/fpsyg.2016.01650

**Published:** 2016-11-03

**Authors:** Marino Pérez-Álvarez, José M. García-Montes, Oscar Vallina-Fernández, Salvador Perona-Garcelán

**Affiliations:** ^1^Department of Psychology, University of OviedoOviedo, Spain; ^2^Department of Personality, University of AlmeriaAlmería, Spain; ^3^Cantabrian Health ServiceTorrelavega, Spain; ^4^Andalusian Health Care ServiceSeville, Spain

**Keywords:** ipseity-disturbance model, self-disorder, schizophrenia, hyperreflexivity, disociation

## Abstract

We know a great deal about schizophrenia, but the current state of the art is one of uncertainty. Researchers are confused, and patients feel misunderstood. This situation has been identified as due largely to the fact that the dominant neurobiological perspective leaves out the person. The aim of the present article is to review and integrate a series of clinical, phenomenological, historical, cultural, epidemiological, developmental, epigenetic, and therapeutic phenomena in support of a suggestion that schizophrenia is above all a disorder of the person rather than of the brain. Specifically, we review seven phenomena, beginning with the conception of schizophrenia as a particular disorder of the self. We continue by looking at its recent origin, as a modern phenomenon, its juvenile onset, related to the formation of the self, the better prognosis in developing countries compared to developed countries, and the high incidence of the disorder among migrants. In the context of these phenomena of a marked socio-cultural nature, we consider the so-called “genetic myth,” according to which schizophrenia would have a genetic origin. On reviewing the current genetic emphasis in the light of epigenetics, it emerges that the environment and behavior recover their prominent role in the vicissitudes of development. The seventh reason, which closes the circle of the argument, concerns the role of interpersonal “chemistry” in recovery of the sense of self.

We know many facts about schizophrenia, but in the end, we do not know what it is (Keshavan et al., 2011). Commenting on Keshavan et al. (2011). Mario Maj highlights the disenchantment and skepticism found today among clinicians and in the general population about the possibility of attaining a robust understanding of the pathophysiology of schizophrenia in the near future, despite so many “facts.” “The huge mass of ‘data’ or ‘evidence’ which is being accumulated in this area,” says Maj, “is not perceived anymore as an indication of a continuing increase of ‘knowledge.’ Rather, this mass of data is increasingly seen as a sign of uncertainty and confusion.” (Maj, 2011, p. 20).

As recognized by Keshavan and colleagues, the current concept of schizophrenia lacks validity, despite its diagnostic reliability. The lack of validity of the schizophrenia concept is “blamed” on its heterogeneity. But perhaps the fact is that heterogeneity is what makes schizophrenia what it is, so that it would be the concept which has to match up to it, rather than trying to reduce it to a collections of symptoms and mechanisms. Nevertheless, the “symptoms” of schizophrenia can and should be understood in their own right, as human psychopathological phenomena, and not merely sub-products of a malfunctioning brain. This is not to say that we should consider possible cerebral anomalies—be they antecedents or consequences of psychotic alteration. The symptoms may be the very expression of a “disordered self,” and at the same time represent efforts to withstand the collapse or crisis itself (Stanghellini and Rosfort, 2015).

Despite the hegemony of the neurobiological perspective, the debate over the nature of schizophrenia remains open (van Os, 2016). This debate is essentially framed in terms of whether schizophrenia is above all a natural condition or a human condition (Pérez-Álvarez et al., 2008b). These two perspectives—neurobiological and psychological—, must be taken into account and combined appropriately, but the crucial question is *how* this is done, giving pre-eminence (chronological, ontological, and epistemological) to one or to the other. Given that it is the dominant conception today, we know what the neurobiological approach has to offer: the dissatisfaction and uncertainty already mentioned.

As far as this article is concerned, we shall argue, with the support of data and sound reasoning, for a consideration of schizophrenia first *and foremost* as a disorder of the person, rather than of the brain. This is a question that cannot be solved on the basis of facts, in terms of Popperian empirical hypotheses. You have to go “beyond the facts,” not to deconstruct schizophrenia as an entity (Nasrallah et al., 2011), but to situate it as a human condition. Schizophrenia considered as a human condition has existential implications for understanding its way of being, as well as scientific with regard to its study from the perspective of human sciences including psychology and psychiatry, more than the neurobiological or biomedical natural sciences. Specifically, we shall put forward seven reasons. Each one refers to an issue under debate and therefore not closed and assigned to the neurobiological perspective, but open to argument in psychological, phenomenological, and cultural terms. The fact that we are talking about matters central to schizophrenia, open and debatable, calls into question the neurobiological approach as an indisputable reference. Each one of these reasons, numbered from 1 to 7, deserves a whole essay dedicated to itself, but here they are presented within the thread of an argument, so that, as it were, the string is worth more than the pearls.

## Schizophrenia as a disorder of ipseity

The conceptualization of schizophrenia presented here mainly follows the developments resulting from the work of US psychologist Louis Sass and Danish psychiatrist Joseph Parnas (Sass and Parnas, 2003), among that of many others. According to this conception, the basic fact underlying the “symptoms” by which schizophrenia is diagnosed is a particular alteration of the experience of the self, or as we shall express it, of ipseity. Ipseity constitutes the pre-reflective bodily self-experience, so that it is also called “basic self” or “minimal self” (Cermolacce et al., 2007; Nelson et al., 2014). Thus, ipseity constitutes the basic structure of the self, whose aspects are sense of ownership and sense of agency (Gallagher, 2007). Sense of ownership is proprioceptive, the pre-reflective experience or feeling that I am the subject who is having the thought or doing the movement (“I am thinking” or “I am moving”). The sense of agency is the pre-reflective experience or feeling in which I am the cause or author of the thought or movement. From this perspective, schizophrenia is conceived as a disorder of ipseity.

An increasing body of literature from recent years is providing a renewed understanding of schizophrenia from this perspective (Bürgy, 2008; Fuchs, 2010; Sass et al., 2011; Henriksen and Parnas, 2012; Parnas, 2012; Akroyd, 2013; Nelson et al., 2014; Parnas and Henriksen, 2014; Sass, 2014; Fielding-Smith et al., 2015; Irarrázaval, 2015; Stanghellini and Rosfort, 2015; Maiese, 2016; Stanghellini and Aragona, 2016). A model of schizophrenia as an ipseity-disturbance has arisen.

### The ipseity-disturbance model

Alterations of ipseity in schizophrenia have three aspects (Sass and Parnas, 2003; Sass, 2014): hyper-reflexivity, diminished self-affection, and alteration of articulation with the world. Whilst the first two aspects involve a disturbance of the normal pre-reflective sense of self as the center of the experience and action, the third one involves the alteration of normal vital contact with reality.

Hyper-reflexivity refers to a type of intensified self-consciousness, in which aspects of oneself that are normally or functionally unnoticed, pre-reflective, tacit or implicit, are objectivized and experienced as objects of consciousness. The reflexivity referred to here is not a type of intellectual, volitional or “reflective” self-consciousness. The hyper-reflexivity characteristic of schizophrenia is an “operative” hyper-reflexivity that occurs automatically, as though the “operating system” of normal psychological functioning were objectivized, no longer remaining tacitly as a background or medium, and became transformed into a figure and an obstacle. This does not mean that schizophrenia does not also involve an intellectual, introspective, metacognitive type of hyper-reflexivity. A variety of bodily experiences occur at the onset of schizophrenia, consisting in experiencing one's own body as an object lived in the third person. Thus, a patient says: “I am no longer myself (…) I feel strange. I am no longer in my body, it is someone else; (…) I hear my voice when I speak, but the voice seems to originate from some other place.” When he does something, he has a feeling of observing his actions as a witness, without being actively involved: “One might think that my person is no longer here (…) I walk like a machine;” (Parnas and Handest, 2003, pp. 126–127).

Diminished self-affection refers to a decline in consciousness of oneself as the subject of experience and of action. There is a diminished self-presence in which the sense of self and the sense of immersion in the world lose their vitality and position. The experience ends up as one of passivity, automaticity, and alienation. The most prominent aspect in the stages preceding the onset of schizophrenia is a type of disturbance in which the self does not saturate experience: “I don't feel myself”; “I am turning inhuman”; “My consciousness is not as whole as it should be”; “I am half awake”; “My I-feeling is diminished.” (Parnas and Handest, 2003, p. 125). The sense of agency, and even of ownership in relation to it being oneself who is having the experiences, are diminished, to the extreme of passivity, automaticity, and alienation.

Alteration of articulation with the world refers to a loss of vital contact with reality. The transformation of ipseity just mentioned (hyper-reflexivity and diminished self-affection) is accompanied by a certain alteration of the objects and field of consciousness; that is, some disruption of the normal configuration of things according to their context. The world appears decontextualized, so that things lose the articulation and context that are taken for granted in accordance with “common sense” and everyday practical life. The world loses its familiarity and context, and one feels strange and perplexed. Others seem dehumanized and devitalized, as though they were not human beings, or only apparently so. This schizophrenic experience is conceived in clinical phenomenology as loss of natural self-evidence or crisis of common sense (Stanghellini J., 2004). Table 1 shows the components of the ipseity-disturbance model.

**Table 1:** **The ipseity-disturbance model**.

**Ipseity**	**Ipseity-disturbance**	**Examples**
Sense of oneself as existing as a vital and self-identical *subject* of experience and action and as a first-person perspective on the world.	Disturbed “mineness”; disorder of self-presence; lack of sense of self.	“Consciousness gradually loses its coherence. The center cannot hold. The “me” becomes a haze, and the solid center from which one experiences reality breaks up like a bad radio signal. There is no longer a vantage point from which to look out, take things in, assess. No core holds things together, providing the lens through which we see the world” (Saks, 2007). “[M]y sense of self is totally crushed when the “bubble” surrounding my self-consciousness is destroyed by this unstable permeability. […] until the entire self-experience disintegrates.” (Kean, 2009).
	Hyper-reflexivity: intensified self-consciousness that involves self-alienation.	Corporeal sensations; de-automatization; thoughts-aloud etc.
	Diminished self-affection: diminished sense of existing as the subject of one's own experience and action.	De-vitalization; feeling influenced; de-personalization.
	“Disturbed hold” or “grip” on the world; alteration of attunement to the world.	Estrangement; de-realization; feeling persecuted; confusion between perception/imagination/memory; uncanny sense of “revelation.”

### Validity of the schizophrenia concept in terms of ipseity

The heterogeneity of schizophrenia, which called into question its established nosological and neurobiological conception (Keshavan et al., 2011; van Os, 2016), ceases to be “surprising” from the perspective of self. Disturbances of ipseity would constitute the “center of gravity” of the diverse symptoms that characterize schizophrenia in its different phases: prodromal, early, and chronic.

In relation to conceptual validity, alterations of ipseity can be shown to be implicated in each of the three major syndromes of schizophrenia recognized in contemporary research: the “positive,” “negative,” and “disorganization” syndromes (Sass, 2003, 2014; Sass and Parnas, 2003; Parnas, 2015). Likewise, deficits of social cognition and of interpersonal relations are understood on the basis of the alteration of ipseity (Nelson et al., 2009; Heering et al., 2016). Contradictions of emotion in schizophrenia between “numbness” or “flat affect” and intense affectivity and hypersensitivity to emotional stimuli can be understood in terms of the alteration of self-experience involved (Sass, 2007; see also Stanghellini and Rosfort, 2013). A phenomenological interview easily highlights the disturbance of ipseity at the bottom of psychotic symptoms. Another thing is understanding the transition from ipseity-disturbance to frank psychosis. From a phenomenological viewpoint what occurs is understood within a process of alienation and externalization or dissociation and depersonalization after the loss of the anchoring center of ipseity (Hirjak et al., 2013):

At the beginning, the patients' impulses and bodily actions are not embedded in the first person perspective anymore. Parallel with motoric fragmentation and hyperreflexivity, drives to carry out actions and bodily movements become disembodied, alienated, and finally externalized. The patients lose control over their body, and its actions now emanate from an external power (Hirjak et al., 2013, p. 7).

In particular, a model based on the dialogical self has been proposed for auditory verbal hallucinations, which understands the voices as dialogical experiences resulting from dissociation of the I-positions (Perona-Garcelán et al., 2015).

As regards discriminant validity, schizophrenia as a disorder of the self-permits better differentiation with respect to bipolar disorder and to normality. Regardless of the symptoms that psychoses may share, studies focusing on subjective experiences reveal basic differences, especially in the domains of self-awareness, articulation of pre-reflective meaning, and perceptual experience (Parnas et al., 2003; Raballo et al., 2011). Whilst schizophrenic people are characterized by, among other aspects, disorders of attunement (“We would need a universal system that covers individual situations”), melancholic people are characterized by orderliness (a need to preserve harmony in interpersonal relations) (Stanghellini J., 2004). If in melancholy one feels imprisoned inside one's body, unable to transcend it, in schizophrenia one does not inhabit the body in the immediate sense, insofar as it is reified (Fuchs, 2005; see also Sass and Pienkos, 2013, 2015).

Alterations of ipseity also improve predictive validity. The most well-established concept for early detection is an “ultra-high-risk” profile: UHR, which includes attenuated psychotic symptoms, transitory psychotic symptoms and risk factors such as schizotypy, family history and anxious and depressive states (Yung et al., 2004). Even though criteria based on UHR represent important progress, they nevertheless present some limitations related to their *superficial* symptomatic nature. After all, predicting psychosis through attenuated or transitory psychotic symptoms is, according to Nelson et al. (2008), like predicting extreme heat through an increase in temperature, without identifying the fire that may be causing this change (p. 384). It is not the symptoms themselves that put an individual at risk, but rather the core or underlying alteration of vulnerability. Indeed, the inclusion of self-disorders together with UHR criteria improves their predictive value (Koren et al., 2016; Raballo et al., 2016). The predictive validity of alterations of subjectivity has also been shown in the detection of subclinical configurations of schizotypy (Raballo and Parnas, 2011). As these authors stress, the “silent side” of the schizophrenia spectrum is the alteration of subjectivity, overlooked until now.

## The modern origin of schizophrenia

Although schizophrenia is usually assumed to be a permanent disorder, inherent to the human being, there are good reasons to claim it as a recent phenomenon (Greenfeld, 2013; López-Ibor and López-Ibor, 2014a,b). Its recent origin would be situated in the 18th century, with an increasing and acknowledged incidence throughout the 19th century. The reasons behind arguments that it is a recent phenomenon are of two types: empirical and conceptual.

### Empirical reasons: notable incidence since the 19th century and a telling absence prior to it

Much of the data and hypotheses about the recent origin of schizophrenia are provided by Edward Hare in his compilation of previous works entitled *On the History of Lunacy* (Hare, 1998) and by Fuller Torrey and Judy Miller in *The invisible plague* (Torrey and Miller, 2007). In reality, Torrey was the first to propose this hypothesis in systematic fashion, highlighting both the growing incidence of schizophrenia in the wake of industrialization and the scarce proof of its existence prior to it (Torrey, 1980).

The first unequivocal description of a case of schizophrenia dates from 1810, According to both Carpenter (1989) and Torrey and Miller (2007, p. 40). This is the case of James Tilly Matthews, admitted to the Bethlam Hospital in London, described by John Haslam in *Illustrations of madness* (Haslam, 1810/1988). As Carpenter (1989) points out, it is the first extensive and detailed description dealing with a variety of anomalous experiences of schizophrenia, among them the “influencing machine” and the apotheosis and reification of the self, which were unlikely to have been described prior to that case. Matthews appears to provide the first example in what would become a long-running theme of “influencing machines,” from the case of Nathalie described by Viktor Tausk in 1919 (Tausk, 1919/1933) to today's cases described by Hirjak and Fuchs (2010). The case of Matthews also seems to be the first reflecting the peculiar experience of the modern self between grandiosity and alienation (Sass, 1992). As the doctors at the hospital reported, Matthews felt “sometimes, an automaton moved by agency of persons, […] at others, the Emperor of the whole world, issuing proclamations to his disobedient subjects, and hurling from their thrones the usurpers of his dominions.” (Haslam, 1810/1988, p. 2). See López-Ibor and López-Ibor (2014b) for the case of Hölderlin.

It is natural for us to wonder whether there might have been some cases of schizophrenia in previous times. In response to this question, it should be stated first of all that if there were any, they were certainly far less frequent than in the period from the 18th century onwards (Torrey, 1980; Hare, 1998; López-Ibor and López-Ibor, 2014a). A merely clinical, symptomatic concept might keep some in the net, though they would still be different in *essence*. Although schizophrenia patients have been reported everywhere, “culture dictates whether and how experiences and behaviors are defined: as schizophrenia-like illness, other type of illness, or non-illness phenomena” (Fraguas, 2009, p. 67).

The case of George Trosse, as told by himself in 1692–1693, is invoked in comparison to the recent hypothesis (Jeste et al., 1985). However, his “symptoms” as shown by Roy Porter, are understood in terms of a “psychomachia” between God and Satan typical of the times, more a religious crisis than a proper crisis of the Self (Porter, 1989, Chapter 5; see also Hare, 1988). With regard to the cases proposed by Heinrichs (2003), one from the 18th century and another from the 14th, the first with persecutory and grandiose delusions and hallucinations, is not out of keeping with the origin of schizophrenia at that time, while the “symptoms” of the second are difficult to see as separate from the religious and allegorical conceptions of the preceding Middle Ages. As Jerome Kroll and Bernard Bachrach justifiably argue: “we find that modern attempts to diagnose medieval mystics and ascetics out of context to their environment and disregarding a commitment to God that constituted the urgent and central feature of their lives is an exercise in cultural insensitivity and conceit. The medieval mystics and ascetics did not have the major forms of mental illness such as schizophrenia and manic-depressive psychosis and their drive to God cannot be explained by recourse to such formulations” (Kroll and Bachrach, 2005, p. 208).

Looking for schizophrenia in Ancient Greece and Rome would also be inappropriate. Epic, tragic, philosophical, or medical madness, according to the different models (Simon, 1978), is not schizophrenia *avant la lettre*, nor should we expect it to be, given that the classical self is not the modern self (Pérez-Álvarez, 2008). For their part, Socrates' voices were voices of reason, not of madness (Leudar and Thomas, 2000). As far as Ancient Rome is concerned, the authors of a systematic review of the literature conclude that: “we were unable to identify any descriptions of chronic psychotic disorders in either fiction or accounts of historical figures that would qualify for a diagnosis of schizophrenia” (Evans et al., 2003, p. 327).

Although the absence of evidence of schizophrenia before the 18th century is not evidence of absence, the discussion of cases brought up and reviews cited support the hypothesis of its recent origin, more than placing it in doubt. In addition, there are conceptual reasons related to the affinity between schizophrenia and modern culture.

### Conceptual reasons: affinity between schizophrenia and modern culture

What is the explanation for the fact that schizophrenia is a relatively recent phenomenon? Everything points to some kind of environmental change. Both Hare and Torrey and Miller suggest a cause of infectious origin. Torrey and Miller refer, moreover, to diet, alcohol, and toxins, as well as the medical care that today saves children susceptible to the illness, but who would previously have died, as potential ways in which the emergence of schizophrenia could be related to industrialization and urbanization. In any case, as they acknowledge, “epidemic insanity remains one of the great enigmas of contemporary medicine and demands novel approaches in thinking about its causes” (Torrey and Miller, 2007, p. 330). A “novel approach” to the historical causes of schizophrenia would involve situating its emergence in the context of modern culture. Industrialization and urbanization are fundamental, but undoubtedly not because of diet, alcohol, toxins, or infections, but rather due to the new way of life they imply (Cooper and Sartorius, 1977; Greenfeld, 2013; López-Ibor and López-Ibor, 2014a).

Two aspects characteristic of modern culture are highlighted. These are the peculiar configuration of the modern self and the drastic social transformation resulting from the process of industrialization and urbanization.

Assuming that the way the self is considered in the society of reference is fundamental to an understanding of schizophrenia (Fabrega, 1989), the modern self would be at the root of this modern origin and character of schizophrenia. We are talking about a self which from the Renaissance onwards has followed an individualistic and internalizing trend. Whilst the individualistic tendency consists basically in a growing separation between the individual and society, the internalizing tendency consists in the separation within the individual of interior and exterior. As shown by Norbert Elias, an invisible wall between one individual and another, and between the self and the universe, as well as between the “internal world” and the “external world” begins to rise up at the dawn of the Modern era (Elias, 2000, 2001). The subject has separated from the object, from the world and from others, and subjectivity has acquired its own reality, to the extreme of even being more real than the world-out-there.

In line with this modern self, thought can become considered as more real than reality, and reality, in turn, as an illusion. Schizophrenia would be the apotheosis of this particular configuration of the self. This duality of consciousness, Sass says, is common to modern thought and to schizophrenia: on the one hand, the solipsism that exalts the mind and derealizes the world, and on the other, the reification of the subject, converted into just one more thing in the world (Sass, 1992, p. 328). As Stanghellini remarks in this regard:

It is legitimate to think that this radical dualism between a subject who is thinking and an object that is conceived of a pure and simple extensive externalness—pure consciousness and pure materialness—is the fundamental *eidos* of both modernity and schizophrenic depersonalization (Stanghellini J., 2004, p. 155).

At the basis of these changes and as a consequence of a whole series of transformations—economic, political, social, religious, etc.—is the great transformation of the society of communities into the society of individuals. In the community, individuals form part and consider themselves members of more extensive structures, such as the immediate family, the extended family, the neighborhood, the village, one's trade, the Church, and so on, against the background of tradition and customs. Without trying to claim that such forms of relation are idyllic and free from conflict, distrust, or whatever, people are certainly not strangers to one another. But the community began to dissolve, and in its place there developed a society of individuals (Elias, 2001), represented by cities from the 18th century onwards, populated by “strangers,” and not in the sense of “people from outside,” but actually “strangers,” “strange people.”

## The juvenile onset of schizophrenia

Schizophrenia is a disorder with onset at the end of adolescence, its peak age in incidence being around twenty. It is no accident that prior to the term schizophrenia, this disorder was considered as particular to youth and referred to as *hebephrenia*, a word coined by Hecker, and inspired by *Hebe*, the Greek goddess of youth. Schizophrenia is linked to adolescence and youth by both its historical origin and its psychopathological developmental roots (Stanghellini G., 2004).

### The historical parallel between schizophrenia and adolescence

The historical coincidence should not be overlooked. As far as schizophrenia is concerned, we have already established its modern origins. As regards adolescence, there have always been adolescents and young people, but as a problematic age range and condition it exists only since the 18th or 19th century (Ariès, 1996). If childhood was a “discovery” of the 18th century, as a distinctive stage (until then the child had been considered an adult in miniature), adolescence would be a discovery of the 19th century, as a space that opened up between childhood and adult life. This *liminal* space represents a critical stage in the formation of personal identity (Erikson, 1968).

The principal consideration about the possible historical affinity between schizophrenia and adolescence has to do with the process of urbanization and what it implies: migration from the countryside to the city and the dissolution of traditional ways of life, as referred to above. Migration from the land to the city and urbanism as a new way of life represent the great transformation, already mentioned, from community to society. Community ways of life are dissolved, and taking their place is a city populated by individuals. Individuals now have to make their way, establish themselves, become something through their own efforts, starting from scratch: family tradition, their own family, the village, their trade, the community—the whole framework of life prior to becoming city dwellers—no longer count for anything (Elias, 2001).

Although migration from country to city also affects children, adults and the elderly, it is reasonable for it to have had a particular impact on adolescence as a critical age in the formation of one's identity and situation in the world. In fact, as Richard Sennett shows in his work *The Fall of Public Man*, “unattached youth” made up most of the growing cities in the decades up to 1750, strangers and “strange people” (Sennett, 1978). It is not being insinuated that the origin of schizophrenia is in migration, but rather in general transformations of modern society such as those mentioned. However, emigration then as now, as discussed further below, could be an especially propitious condition for a psychotic crisis, not to mention other disorders. It is not a coincidence that at that time a “new” disorder began to be observed, which not by coincidence either, was called “hebephrenia.”

We have no demographic data or personal case histories to tell us who the “hebephrenics” were and what their life circumstances were like. Hecker himself, indeed, complained that these patients arrived at the hospitals already in late stages of the illness. In any case, it can be conceived of as a crisis related to the difficult balance between the self and the world. The first case described by Hecker, from 1871, appears more a response to life circumstances than the consequences of biology. The case reported by Hecker is of a 20-year-old who talks to himself, remains apart from others, and laughs or becomes angry for no apparent reason. He is evasive in his answers. For example, to the question “‘How are thing's going?’, he mysteriously replies ‘Well. You need to at least have your own freedom.’ He's disobedient, contrary, bothersome, and quarrelsome […] and answers all questions with these enigmatic words: ‘But my eyes! But my eyes!”’ (Stanghellini G., 2004, p. 473). Nothing is known about this disorganized behavior, and it may be on a one-way road, whereby the behaviors themselves gradually may constitute *feedback loops* in which the possibilities of life become narrowed, leading down a path to a development disorder in continuation.

In the absence of the data that would be useful here, it might make sense to consider current phenomena that undoubtedly correspond, in some measure, to what occurred in the 18th and 19th centuries. We refer to the high incidence of schizophrenia among current immigrants from traditional communities in European cities (which would constitute the fifth reason), as well as the better prognosis for schizophrenia in developing countries (the fourth reason for consideration).

### Schizophrenia as a developmental disorder

The neurodevelopmental model is well established (Catts et al., 2013; Haller et al., 2014). Two critical periods for prefrontal cortical development have been described, early prenatal and adolescence. A wide spectrum of environmental factors impinging on the brain during these critical periods can modify the trajectory for prefrontal cortical development and shift the balance toward mental illnesses such as schizophrenia (Selemon and Zecevic, 2015). Many studies have demonstrated high levels of cytokines and other signs of immune system activation. Furthermore, cytokines are critically involved in early neurodevelopment and deviations from the norm can result in abnormal neuroanatomy and brain chemistry (Howard, 2013; Ratnayake et al., 2013).

The meaning of the data depends on how schizophrenia is theorized. According to Catts et al., “When theorizing about the neurodevelopmental basis of schizophrenia, schizophrenia could be considered to result from a failure to reach the final state of cortical maturation resulting in retainment of an immature cortex (at least transiently)” (Catts et al., 2013, p. 18). However, it may also be theorized based on psychodevelopment within the framework of a conception of schizophrenia as an ipseity disorder. On one hand, it would be the gulf between molecular conditions and the schizophrenia experience, a distance still greater than the one recognized by Freud between the Oedipus complex and killing one's father. On the other, it would be the decisive role of the environment beginning with the interpersonal role as etiopathogenic and restorative. In this respect, Catts et al refer to the psychosocially informed approach of McGorry (2011) for “stabilize the adolescent brain” (Catts et al., 2013, p. 18). Finally, it would be the evidence itself and theorization from a sociodevelopmental perspective which includes neurodevelopmental factors. But the neurodevelopmental perspective also includes psychosocial factors. The question lies in whether one gives primacy to the formation of the brain or the formation of the self. These two “formations,” we need hardly add, are mutually implicated, and must be considered thus.

However, there are good reasons for approaching the issue from the psychodevelopmental side. For a start, the basic fact that schizophrenia, as we have argued, is a certain alteration of the self. And as is well known, adolescence is a *critical* period in the formation of the self. It is not a case of leaving out the brain, but neither of putting it first, as the neurobiological perspective does. The brain itself may be modulated, thanks to its plasticity, by culture, life circumstances and efforts to adapt to the world (Toyokawa et al., 2012). So as not to incur in the usual neurocentric explanation, the interactive brain hypothesis is claimed here as a mediating organ of (Fuchs, 2011; Di Paolo and De Jaegher, 2012), neither causal nor creating, according to an embodied, embedded and enactive approach. Contrary to the neurocentric and individualist approach,

the enactive approach foregrounds a different notion of the living body (of which the brain is a part) in its ongoing sense-making relation to the world. According to this approach, the brain is understood as embedded, not in a protective and nourishing casing, but in ongoing circular processes of sense-making that pass through it, the body and the world; in other words it is understood as a mediating organ (Fuchs, 2011) with all the implications that this view has for the study of brain function (Di Paolo and De Jaegher, 2012, p. 13).

There is a well-documented affinity between certain characteristics of adolescence and schizophrenia (Harrop and Trower, 2003). These would be normal characteristics of adolescence, such as feeling special and unique, and an intensified self-awareness. Likewise, quasi-psychotic experiences are common in adolescence, akin to those found in schizophrenia—thinking that the things coming out of the TV have special significance for oneself, believing that people can read other people's minds, and so on (Fonseca-Pedrero et al., 2011). In this sense, schizophrenia could be understood as an exaggerated, pathological form of adolescent experiences. Although the vast majority of adolescents do not *attain* schizophrenia, it is also true that the majority of cases of schizophrenia have their beginnings in adolescence.

In line with Harrop and Trower (2003), it is understood that those who develop schizophrenia have become bogged down in some way during adolescence. This may occur when there is a lack of individuation with respect to one's parents, or when the adolescent fails to forge bonds with peers. In either case, the adolescent will become bogged down in a whole range of disturbances, among them psychotic-type experiences.

### Adversities, dissociation, and schizophrenia

Beyond such situations of stagnation in adolescence, it is necessary to consider patterns of attachment and traumatic experiences. The theory of attachment permits an understanding of the relation between types of interpersonal events and the development of adult disorders (Read and Gumley, 2008). For its part, trauma is practically the sole recognized cause of schizophrenia, despite not being specific to this disorder, nor probably at the origin of all cases (Read et al., 2005; Morgan and Fisher, 2007). Likewise, a growing body of research consistently shows that dissociation is a mediating variable between, on the one hand, disorganized attachment and trauma, and on the other, psychotic symptoms (Moskowitz, 2011; Perona-Garcelán et al., 2012).

In terms of neurophysiological mechanisms, dysregulation of the hypothalamic-pituitary-adrenal (HPA) axis is probably implicated in schizophrenia (Howes and Kapur, 2009). It is understood that sensitization of the HPA axis is the common final route, so that repeated exposure probably increases the behavioral, cognitive, experiential, and neurophysiological responses to the new stress, with the possible consequence of increasing risk for psychotic experience. In this line, it is conceivable that there is involvement of neurobiological mechanisms and epigenetic changes, in response to the impact of one's experiences and of a pressurizing environment (Toyokawa et al., 2012).

**Figure 1:**
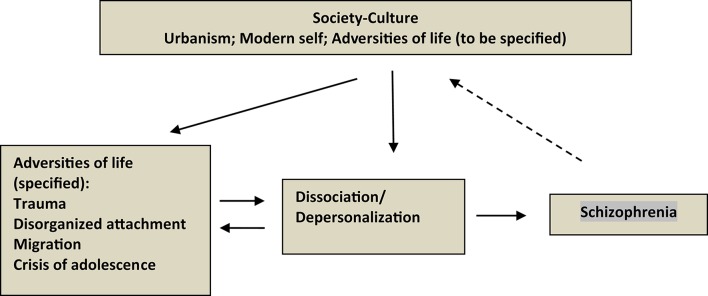
**Schema of the Sociodevelopmental Model of Schizophrenia**. The model is characterized by the mediation of dissociation/depersonalization between life adversities and the symptoms that define schizophrenia. It is understood that between the adversities of life and dissociation/depersonalization there is mutual influence, and that, in turn, schizophrenia can be one more adversity. The three terms of the model are situated against the background of modern culture-society, whose notable aspects are urbanism as the most characteristic medium of modern life, the peculiar configuration of the self, with its autistic (“schizoid”) tendency, and the notion of life adversities, in whatever form.

If we accept that the adversities of life have chronological and etiological priority in the development of schizophrenia, through psychological alterations (such as dissociation) and the corresponding neurobiological mechanisms (e.g., sensitization of the HPA axis), we can propose a sociodevelopmental model, alternative to the current *neurodevelopmental one* (Read et al., 2009; Morgan and Hutchinson, 2010a; Read, 2013). Figure 1 schematizes this sociodevelopmental model. What the figure is designed to show is that the dissociation/depersonalization emerges in the theory and in research as the mediation between the adversities of life and the alterations that characterize schizophrenia. The three terms of the model, adversities—dissociation—schizophrenia, are situated against the background of modern culture-society.

## Better prognosis for schizophrenia in less developed countries

Schizophrenia has better prognosis in developing countries than in the developed world. This is a surprising but consistent finding, and among those reported in studies sponsored by the World Health Organization (WHO). Three large studies have been carried out (Sartorius, 2007): *International Pilot Study of Schizophrenia (IPSS*), *Determinants of Outcome of Severe Mental Diseases (DOSMeD)*, and *International Study of Schizophrenia (ISoS*).

### Robust and sustainable finding

“For all variables considered, the schizophrenic patients in Ibadan, Agra, and Cali (all centers in developing countries) tended to have a better outcome on average than the schizophrenic patients in the other six centers” (Sartorius, 2007, p. 5). This result from the first study (IPSS) was repeated in the following studies. Thus, referring to the second study (DOSMeD), a significantly higher percentage (56%) of the subjects in developing counties exhibit “mild” patterns of course, compared to their counterparts in developed counties (39%). At the same time, a significantly higher percentage (40%) of the cases in developed countries had “severe” patterns of course, compared to the cases in developing countries (24%). The third study (ISoS) confirms the previous findings (Sartorius, 2007).

The fact of the existence of schizophrenia in developing countries does not necessarily contradict the thesis about its recent origin in the context of modern society. As *developing* countries, with Western-style psychiatric attention centers, these countries are modern to some degree, and in the same proportion appear to have schizophrenia, *not yet developed*, perhaps due to the persistence of community structures.

### What do less developed countries have that produce a lower incidence of schizophrenia?

Whatever it is, it probably has to do with the different ways of life found in a *traditional* community-based society, in contrast to a *modern* society of individuals, as discussed already in relation to the recent origin of schizophrenia. As Cooper and Sartorius (1977) argue, the current situation of developing countries may be similar to that of many Western countries during the great transformation of pre-modern into modern society. In this line, Lin and Kleinman (1988) highlight five conditions that can explain the better prognosis for patients in non-industrialized countries, referring to social support, the family environment, the nature of work, stigma, and the differential survival rates of vulnerable individuals. In any case, over and above any lists of characteristic social factors, it would be necessary to carry out anthropological studies revealing everyday ways of life in relation to those who have psychotic access, such as that of Juli McGruder in Zanzibar, Tanzania (McGruder, 2004). From McGruder's study, which resulted from her living with various families with schizophrenic members, three aspects should be highlighted (McGruder, 2004; Watters, 2010).

One of these is that the idea that people in traditional cultures have a simpler and less stressful life is just a fantasy in the heads of Western people. The toughness of life, social conflicts, wars, family problems—they are far from free of such challenges. The supposed lack of or lower level of stress is not what is behind the more “benign” schizophrenias found there. Another aspect is the *social-moral status* of the member of the family who is ill, as opposed to a particular-individual status, which precludes criticism and hostility. If the illness has to do with “tests from God” (or Allah) and possession by spirits that form part of the universe of that community, it becomes normalized in the broad context of the family. The third aspect is the normal acceptance and tolerance of the ill person. It is a naturalized tolerance, described by McGruder in terms of acquiescence in the face of adversity and the embracing of difficulties as part of life (McGruder, 2004).

In this context it is understood that mental illness has less stigma attached to it than it would in a medical context. As Watters (2010) remarks in relation to McGruder's study, what could be more stigmatizing than reducing a person's perceptions and beliefs to the notion that they are “just chemistry?” This is a narrative that often drives a person out of the group, allowing those remaining within the social circle to see the ill person as “almost a different species” (Watters, 2010, p. 195).

## The high incidence of schizophrenia among migrants

High rates of schizophrenia have repeatedly been found over the last 20 years among immigrants from various countries in a range of European cities (Coid et al., 2008). It is now emerging a similar problem with refugees (Hollander et al., 2016).

### A challenging phenomenon for psychiatry

Quite apart from the public health tragedy involved, this phenomenon represents quite a challenge for psychiatry (Morgan and Hutchinson, 2010b). Its impact for psychiatry concerns the aetiology, pathophysiology and treatment of schizophrenia, and hence its conception and scientific and clinical status. This high rate of schizophrenia, within its variability in the different studies, is between 2 and 8 times that for the indigenous British or Dutch populations. At the same time, it is high in the same proportion with respect to its incidence in the populations of their countries of origin. The finding is robust and consistent, and is not subject to methodological artifacts, since the studies have been repeated and improved and the reviews are increasingly exacting (Coid et al., 2008; Morgan and Hutchinson, 2010b).

However, not all immigrants in Europe show high rates of schizophrenia. This phenomenon is notable among migrants from the West Indies, in particular African-Caribbeans, and also among Moroccans, but this does not seem to be the case among migrants from Asia or Turkey. The incidence of schizophrenia among Asian immigrants in London is lower than that for other groups, and similar to that for the populations of their destination and origin (Chang et al., 2011), even though levels among Asian women are increasing (Coid et al., 2008). Likewise, the incidence of schizophrenia among Turkish immigrants in Holland is lower than it is among other groups (Veling et al., 2007).

Various explanations are put forward for the findings. The self-selection explanation often proposed, according to which migrants would be those with some predisposition to psychosis, together with explanations based on genetic and neurobiological causes, have been discredited by the data (van der Ven et al., 2015). It is the turn of an explanation in environmental terms, be they biological, social or an interaction of the two. Among the biological factors investigated, no increased prevalence has been found for birth complications or neurobiological abnormalities diagnosed; as far as drug abuse is concerned, rates are not significantly higher among migrants (Morgan and Hutchinson, 2010b). Other “suspects” would be vitamin D and epigenetic mechanisms. There remain the social factors.

### Social causes of schizophrenia

A number of social factors are involved: urban stress, unemployment, poverty, family separation, racial discrimination, and so on. Two explanations have been proposed: social defeat theory and the sociodevelopmental model, already introduced in our discussion of the juvenile onset of schizophrenia.

The theory of social defeat refers to a particular pattern of social interaction whereby people are the victims of disdain, humiliation, and subordination from others (Selten et al., 2013). Social defeat theory integrates social, psychological and biological mechanisms (in that order) to account for the development of psychotic symptoms. In accordance with this proposal, continued and chronic experiences of social defeat imply a sensitization of the mesolimbic dopamine system and/or increased basal activity of this system, and hence a higher risk of schizophrenia, insofar as the dopaminergic system appears to be involved in it.

The sociodevelopmental model emerges in relation to the abundant and robust evidence showing the link between not only migration, but also a broader range of experiences and social and psychological factors (trauma, social adversity), and the onset of psychosis (Morgan and Hutchinson, 2010a; Morgan et al., 2014). Thus, it has been seen how repeated exposure to social adversity can be linked to psychosis through delusional ideas, as well as the processes of dissociation and depersonalization mentioned above (Perona-Garcelán et al., 2012).

And what of the lower incidence of schizophrenia observed among Asian and Turkish immigrants in European cities? These differences in favor of Asians and Turks probably have to do with the fact that their system of emigration incorporates the family and their own customs wherever they go (Veling et al., 2007; Coid et al., 2008). They maintain the community at their destination, so to speak, in contrast to the case of “solitary” individuals in other systems of migration. For example, Turks in The Hague perceive less discrimination and show lower incidence of schizophrenia than Moroccans, even though they have similarly low economic status (Veling et al., 2007). Similar regarding to Asians:

It has been suggested that the more cohesive cultural, ethnic, and religious structure of Indian, Pakistani, and Bangladeshi communities may confer greater social support than in other groups that may otherwise share similar levels of discrimination. That the excess risk of psychoses for Asian immigrants in our sample appeared to be restricted to women provides anecdotal support for the social defeat hypothesis given the additional pressure of marginal status faced by some women in Indian, Pakistani, and Bangladeshi communities (Coid et al., 2008, p. 1256).

If we are to take culture seriously—and it is high time we did—it is these types of aspects (emigration systems, social integration, etc.), that we need to study, no less than the genes, chromosomatic alleles, and neurodevelopmental trajectories.

## The genetic myth of schizophrenia

There is, one might say, a genetic *myth* in relation to schizophrenia, whereby authors speak of 80% heritability, a concordance of 40–50% between monozygotic twins and a worldwide prevalence of 1% (Gottesman, 1990; Sullivan, 2005; van Os and Kapur, 2009; Keshavan et al., 2011). However, there are also serious objections that should not be overlooked before accepting this “genetic consensus.”

### Statistical genetics and “real” genetics

To begin with, the epidemiological, family, twin, and adoption studies on which this myth is based do not adequately support such assertions (Leo, 2003, 2016; Crow, 2008; Fleming and Martin, 2011; Joseph, 2013; James, 2016). Both the methodological problems of these studies and the intricate nature of the very relationship between genetics and environment preclude the establishment of percentages and causal relations, as well as statements such as “schizophrenia is due mainly to genetic effects” (Sullivan, 2005, p. 615). Thus, for example, in relation to the supposed concordance of 40–50% between monozygotic twins raised separately, quite apart from the fact that this reveals both genetic and environmental influences, careful analysis of the data shows that the concordance would actually be of the order of 15–25% (Leo, 2003; Joseph, 2013). However, and as these authors stress, the question is not the accuracy of the percentage, but rather the inextricability of genetics and environment, insofar as genetics itself is modulated by the environment even while we are in the womb.

The proposal of this argument is not to deny the heritability of schizophrenia, but rather to insist that we cannot make assertions in either percentage terms or strictly genetic terms, given the need to take into account, as well as genetic transmission, epigenetic, behavioral, and cultural transmission (Jablonka and Raz, 2009; González-Pardo and Pérez-Álvarez, 2013). The fact that schizophrenia runs in the family does not mean that it is in the genes; after all, religion and accent, for example, also run in the family. Furthermore, the passing on of schizophrenia via the family would be surprising, seeing as these individuals are by no means the most prolific reproducers (Hare, 1998, chap. 6), and sporadic cases, with no family antecedents, account for at least half of the total.

“Real genetics,” with a molecular basis, has not done justice to the optimism of “statistical genetics” by detecting major gene effects in schizophrenia (Gottesman et al., 1987, p. 41). In 1987, these authors were confident that molecular genetics would confirm the statistical genetics. While the human genome has been described and ground-breaking methods and projects have been developed, the genes for schizophrenia are still nowhere to be seen. From the most comprehensive genetic association study of genes previously reported to contribute to the susceptibility to schizophrenia, based on reasonable candidate genes, a large, rigorously phenotyped sample and a dense set of single nucleotide polymorphisms (SNPs) (Sanders et al., 2008), what did the authors find? That “none of the polymorphisms were associated with the schizophrenia phenotype at a reasonable threshold for statistical significance” (Sanders et al., 2008, p. 504).

Although the disappointing study focused on candidate genes may possibly continue, genetic research is adopting the genome-wide association studies (GWAS) paradigm. In contrast to gene-specific candidate-driven studies, GWAS investigate the entire genome, in which hundreds of thousands of SNPs are tested for association with a disease in hundreds or thousands of persons. GWAS have revolutionized the search for genetic influences on complex traits, identifying hundreds of genetic variants that contribute to a variety of common traits and illnesses. But even though dozens of genes have been linked to a trait or disease, the individual and cumulative effects are disappointingly small, and far from sufficient for explaining the previously estimated heritability (Manolio, 2010). Larger and larger samples are showing smaller and smaller effect sizes (James, 2016; Leo, 2016).

This disappointing finding has turned the issue of personal genomes into the case of the missing heritability, a veritable mystery (Zuk et al., 2011). The case of missing heritability highlights the discrepancy between “statistical” genetics, on which the genetic myth is based, and “real” genetics, in the wake of the sequencing of the human genome. There are several possibilities. A possibility is that the missing heritability consists, partly, in copy-number variations (CNVs), emerging *de novo*, in an individual without family history of mutation (Bassett et al., 2010; Xu et al., 2011). However, and despite the enthusiasm with which this strategy has been received, the truth is that CNVs account for no more than 2% of schizophrenia in the general population (Bassett et al., 2010). In reality, the majority of the genetic variants so far identified (Sekar et al., 2016) confer very small increments in risk of schizophrenia from 1.0 to 1.27% (Ross, 2016).

A further possibility is that such or so much heritability simply does not exist, because it is over-estimated. Not to mention the general public, the readers of scientific journals themselves are “lured by the thesis that (1) a major thrust of psychosis research is now genetic, (2) there is substantive progress, and (3) genes that contribute to predisposition have already been identified.” (Crow, 2008, p. 1681; Joseph, 2013; James, 2016; Leo, 2016; Ross, 2016).

### From genetics to epigenetics: a prominent role for the environment and psychological experiences

The genetic enthusiasm intensified by the advent of GWAS has tended to overshadow the decisive role of environment in the acknowledged G-E interactions, so that “better ontologies” are needed (Thomas, 2010). In general, the G-E interaction tends to be understood in accordance with a linear model, in which the origin of the disorder is situated in the genome, waiting to be “activated” by the environment (van Os and Poulton, 2009, p. 57). This gene ontology reflects the “looking under the lamp post” mentality, whereby bioinformatics data are easier to recruit (Thomas, 2010). An authentic “gene ontology” must tackle the full and complex reality, including the bidirectional, non-linear relations between, on the one hand, genetic components and cellular contexts, and on the other, environmental components, which would include experiential, behavioral, social and cultural factors. As we now know, what is decisive in human genetics is what occurs above (*epi*), between the zygote and the development of the organism, in a sociocultural context, studied by epigenetics (González-Pardo and Pérez-Álvarez, 2013). As more is known about the genetics of schizophrenia, environmental factors appear to be the most important (Stepniak et al., 2014).

**Figure 2:**
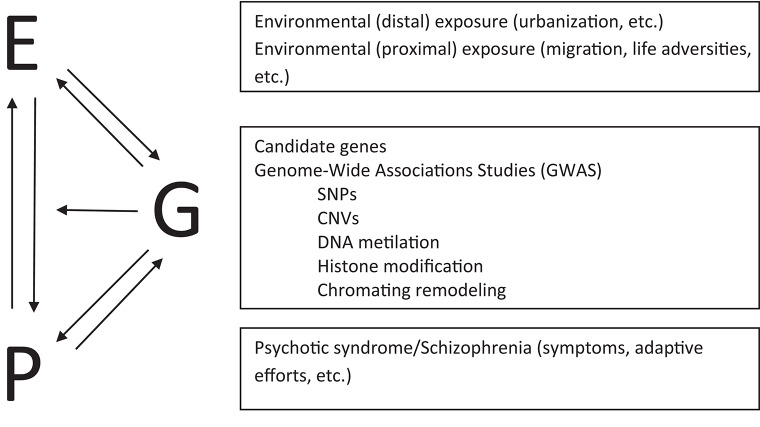
**Model of interplay between Environment (E), Genotype (G), and Phenotype (P)**. The environment can shape phenotypes as well as inducing epigenetic changes. Phenotypes, that is, people's psychotic symptoms and adaptive efforts, can build environments which in turn induce and select epigenetic changes. Although phenotypes are never separated from the environment, we can conceive of their direct epigenetic influence, as way of life or continued pattern of response. Genetic dispositions can select environments and influence phenotypes, which in turn feedback influence in terms of genetic stability. In any case, what is involved is an ongoing and intricate interplay: environment-behavior-genetics. SNPs, single nucleotide polymorphisms; CNVs, copy-number variations.

Epigenetics shows how there is environmental regulation of the genome and its functions, whereby the different genomic, contextual, and environmental components interact non-additively throughout development (Gottlied, 2000). The behavior of organisms themselves, in this case the phenotype, influences the genome, both through its influence on the environment and directly. We are identifying the epigenetic and neuronal mechanisms through which behavior and the social environment “get into the mind” (Toyokawa et al., 2012). Thus, in relation to schizophrenia, it has been possible to identify epigenetic mechanisms such as DNA methylation, histone modifications, and chromatin remodeling (Rutten and Mill, 2009). What is important to highlight here is that these epigenetic changes occur in response to life adversities known to be associated with schizophrenia, such as abuse in childhood (Read et al., 2009; van Os et al., 2010; Brown, 2011). Possible vulnerability to future experiences due to early epigenetic changes should be understood in the context of original and continued life adversities, according to bi-directional causality (Gottlied, 2000; Laland et al., 2011). The linear G-E model should give way to one of reciprocal influences that incorporates the phenotype (van Os et al., 2010). Figure 2 shows a schema of this model.

## A new lease of life for the psychotherapy of schizophrenia

In the words of a patient: “People talk about chemical imbalance. Well, [my friend] and I, [together we made] a chemical balance” (Davidson, 2003, p. 170). However, the treatment of choice for schizophrenia is so-called antipsychotic medication. Notwithstanding the fact that it would be difficult to dispense with it, the truth is that antipsychotic medication presents at least three problems as the treatment of choice: (1) It is merely symptomatic, insofar as it ignores the circumstances that have led to the crisis. (2) The fact of starting with medication can “mark” the destiny of the chronic patient. Medication becomes the topic of subsequent visits—whether to maintain it, reduce it, change it, etc., as part of a process called “listening to the drug,” instead of to the people themselves. (3) Medication difficult the course of authentic psychological therapy. Thus, the goal of psychotherapy is not necessarily to eliminate the symptoms, but rather to change the relationship with them and develop an understanding of their meaning (Pérez-Álvarez et al., 2008a).

### For a phenomenologically-oriented psychotherapy

We propose an approach of phenomenologically-oriented psychological therapy, in whose framework other types of therapies can be acknowledged and integrated (Pérez-Álvarez et al., 2011). The emphasis on the phenomenological perspective stems from the goal of attempting to reconstruct the integrity of the self which, in accordance with the conception outlined, would be essential for repairing the alterations that characterize schizophrenia (Irarrázaval and Sharim, 2014; Vallina-Fernández et al., 2014).

The condition *sine qua non* in the psychotherapy of schizophrenia is a particular kind of therapeutic relation. This interpersonal encounter would be more focused on an understanding of the person's altered being-in-the-world experience than on repairing the supposed malfunctioning of a mechanical system (Nelson et al., 2008, p. 283). Phenomenology offers a vision that situates patient's disorders not in the hidden circuits of their brains, nor in remote corners of their minds, but in the real word of their lives with others, in the *Lebenwelt*, which is, after all, the only world in which psychotherapy takes place (Fuchs, 2007).

An empathic bridge between the therapist and the patient is at the basis of possible recovery in schizophrenia. The space necessary for a person to emerge from the illness is opened up by means of the person being perceived by others as more than his or her illness (Davidson, 2003, p. 173). Stanghellini and Lysaker (2007) have shown that this occurs in psychotherapy sessions, when the therapist repeatedly offers a second-person view, when one “you” addresses another “you,” as opposed to a third-person view referring, for example, to the “illness” or to some supposed mechanism of it, such as an underlying conflict or a cognitive process. These authors show through vignettes of therapeutic sessions that the “inter-subjective method” can help people with schizophrenia to develop the first-person perspective for themselves and the second-person perspective when they are with others, thus opening up the way to recovery.

Within this approach, the narrative takes on special importance (Roe and Lysaker, 2012). The “narrative turn” involves listening to patients and taking seriously their experiences and “stories.” Patients' recovery, as they themselves report, is characterized by a renewed sense of hope, the understanding of their own experiences, active participation in life, the resurgence of a sense of personal responsibility, and the view of oneself as a person (Riggay, 2001).

More specifically, the narrative can serve as a thread for re-authoring lives. What is behind the symptoms is a tormented, disconnected, disintegrated, diminished self. As one patient says, “[S]chizophrenia is ultimately a disorder of the self, a disturbance of one's subjective self-experience and the external or objective reality” (Kean, 2009, p. 1). Therapeutic narratives make sense of psychotic experiences, explaining the “symptoms” in the biographical context, linking affects and cognitions, confronting life events, proposing alternatives, opening horizons, etc. Narratives do not merely recount experience, but actually form it, insofar as the functions of language and hermeneutics are not only expressive, but also constitutive of one's own experience and personal history (Raffard et al., 2010; Stanghellini and Rosfort, 2013).

### Not just narrative, but also acceptance and action

Acceptance is an active attitude of understanding-based self-distancing, not of passive resignation, vis-à-vis uncomfortable experiences that one had hitherto tried to avoid or control, but with the paradoxical result of exacerbating the distress in the long term. Acceptance is important in schizophrenia in relation to upsetting experiences, such as voices, the attempt at control of which is often more pernicious than beneficial. As opposed to the fruitless attempt to eliminate the voices, a better objective is to change one's relation with them, one strategy being acceptance (Pérez-Álvarez et al., 2008a, 2011). Acceptance has much to do with mindfulness, a more common application in cases of psychosis (Abba et al., 2008).

For its part, commitment consists in reorientation toward values significant for one's life, despite the fact that certain experiences persist and disturb. Commitment is acting in the direction of values in spite of the distress caused by the symptoms. The question is not to wait until one gets better before going out and living, but rather to act and remake one's life so as to feel better, or at least, to be on the way to something, and not just shut up inside oneself. This reorientation of self-experiences toward values begins with the identification and clarification of one's own values. From the phenomenological point of view, the reorientation of life to values, rather than to self-experiences, may serve to reduce the hyper-reflexivity and intensified self-awareness that characterize schizophrenia (Pérez-Álvarez et al., 2011; Fuller, 2013; Maiese, 2016).

## Conclusions: closing the circle

We have proposed seven reasons, linked together, for a reconsideration of schizophrenia first *and foremost* as a disorder of the person, not of the brain. The argumentational thread is the role of the self, of the subject or of the person in the disturbance for which schizophrenia is diagnosed. This thread is at the basis of each one of the reasons discussed.

The first reason deals with the actual conception of schizophrenia as a disorder of the experience of oneself and of the world (*ipseity*), in accordance with criteria of a phenomenologically-informed psychopathology (Fuchs, 2010; Stanghellini, 2010). Schizophrenia being an alteration of the basic self or center of gravity, it is understood as affecting the person *in toto* and his or her way of being-in-the-world (Sass, 2014; Stanghellini and Rosfort, 2015). The second reason refers to its modern origin. The relevance of this reason lies not only in the notable incidence of the disorder found after 1750 and glaring absence before then (Torrey and Miller, 2007; López-Ibor and López-Ibor, 2014a), but also in the substantive affinity between the modern self and the alteration characteristic of schizophrenia (Sass, 1992; Stanghellini J., 2004).

The third reason concerns the juvenile onset of schizophrenia, which has a dual implication in the perspective taken here. One is that adolescence, the period in which schizophrenia usually has its beginnings (Harrop and Trower, 2003), is a critical time in the formation of the self (Erikson, 1968). Thus, certain normal characteristics of adolescence have aspects in common with the clinical manifestations for which schizophrenia is diagnosed (Fonseca-Pedrero et al., 2011). The other implication is that adolescence as a critical age is a historical phenomenon that coincides with the modern origin of schizophrenia, so that it is no coincidence that the name of the disorder *avant la lettre*—hebephrenia—denoted youth (Stanghellini G., 2004). The fourth reason, the better prognosis for schizophrenia in developing countries compared to those of the developed world, may also have to do with the role of modernization in the determination and configuration of schizophrenia (Cooper and Sartorius, 1977).

The fifth reason, referring to the high incidence of schizophrenia observed among migrants from traditional communities such as the former colonies of the West Indies in European cities, at the same time as calling into question the well-rehearsed genetic and neurobiological explanation, demands an alternative one in terms of social causes (Morgan and Hutchinson, 2010b). Moreover, this phenomenon could be linked in with the high incidence of schizophrenia observed in “modern times,” in line with the content of the second reason. The exposition of the sixth reason includes a reappraisal of the role of genetics in schizophrenia. Without negating its possible role, but not magnifying it either (Joseph, 2013; Ross, 2016), here genetics is not given primacy, but rather situated in the context of the conditions of life and of development, in accordance with epigenetics, whereby the decisive aspect in genetic terms is what occurs over the whole course of development. Thus, environmental factors such as abuse as a child, disorganized attachment or the impact of emigration, may bring about epigenetic changes that predispose one to schizophrenia (Read et al., 2009; Rutten and Mill, 2009). Schizophrenia may indeed be hereditary, without being genetic (Jablonka and Raz, 2009).

The seventh reason, which closes the circle, is the possibility of a psychotherapy of schizophrenia, without detriment to its being complemented by drugs where necessary. The sensitivity of schizophrenia to psychological therapy is understood in accordance with the interpersonal context provided, a condition for the recovery of one's sense of self (Fuchs, 2007; Stanghellini and Lysaker, 2007). If, as stated in our exposition of the first reason, schizophrenia stems from a crisis of the sense of self, a natural recovery involves the provision of an interpersonal context such as that of psychotherapy (Davidson, 2003). The possibility of a psychotherapy of schizophrenia is based on the simple but fundamental idea that people diagnosed with schizophrenia *are* people, and continue to be so.

The reasons considered situate schizophrenia as a human condition—rather that a natural one—of historical-cultural origin and biographical nature, related to vicissitudes and circumstances of life. In any case, the nature of schizophrenia is and will continue to be controversial and for that very reason is worth a proposal such as the present one, which does not lessen the possibility of future improvements.

## Author contributions

The hypothesis and theory derives from all authors as a research group. MPA drafted the manuscript. All authors edited the manuscript, read it and approved the final versión.

### Conflict of interest statement

The authors declare that the research was conducted in the absence of any commercial or financial relationships that could be construed as a potential conflict of interest.
